# Supplementary material for: High-efficiency CRISPR gene editing in C. elegans using Cas9 integrated into the genome
Source: PLoS Genet. 2021 Nov 8;17(11):e1009755. doi: 10.1371/journal.pgen.1009755 (PMC8601624; doi:10.1371/journal.pgen.1009755)
Supplement: S1 Table — Number of injected P0 worms (EG9747) that yielded at least one GFP insertion event on primary or secondary plates. ‘Primary + Secondary’ tallies P0s for which GFP+ worms were found on both the primary plate founded by the single injected P0 and on the secondary plate founded by 20 array+ GFP- progeny of the originally injected P0 (~F3). ‘Primary Only’ tallies P0s for which GFP+ worms were found on the primary plate but not on the secondary plate. ‘Secondary Only’ tallies P0s for which GFP+ worms were not found on the primary plate but were found on the secondary plate. ‘Neither’ tallies P0s for which GFP+ worms were not found among the progeny on either the primary of secondary plate. (PDF) [file pgen.1009755.s001.pdf]

Gene	Primary + Secondary	Primary Only	Secondary Only	Neither	Total
sng-1	1	4	1	2	8
snb-1	5	0	0	0	5
rab-3	6	3	1	5	15
unc-32	4	3	0	1	8
unc-17	19	0	0	0	19
snt-1 (short)	0	0	0	6	6
snt-1 (long)	4	2	2	13	21

S1 Table Post-silencing edits. Number of injected P0 worms (EG9747) that yielded at least one GFP insertion event on primary or secondary plates. 'Primary + Secondary' tallies P0s for which GFP+ worms were found on both the primary plate founded by the single injected P0 and on the secondary plate founded by 20 array+ GFP- progeny of the originally injected P0 (~F3). 'Primary Only' tallies P0s for which GFP+ worms were found on the primary plate but not on the secondary plate. 'Secondary Only' tallies P0s for which GFP+ worms were not found on the primary plate but were found on the secondary plate. 'Neither' tallies P0s for which GFP+ worms were not found among the progeny on either the primary of secondary plate.
