# Supplementary material for: High-efficiency CRISPR gene editing in C. elegans using Cas9 integrated into the genome
Source: PLoS Genet. 2021 Nov 8;17(11):e1009755. doi: 10.1371/journal.pgen.1009755 (PMC8601624; doi:10.1371/journal.pgen.1009755)
Supplement: S2 Table — Summary of analyses of 14 Cas9 miniMos integration strains. ‘Strain’ is the isolation name. ‘chrom’ and ‘gene’ identify the location of the insert determined by inverse PCR, ‘unmapped’ means the inverse PCR failed or was ambiguous. PCR confirmation: ‘+’ indicates position was confirmed by locus-specific PCR, ‘-‘ indicates confirmatory PCR failed. ‘n.t.’, not tested. ‘cuts dpy-10’: P0s injected with a dpy-10 sgRNA expressing plasmid, ‘+’ indicates the presence, and ‘-‘ indicates the absence of Dpy and Rol worms among the progeny of those P0s. ‘GFP inserts’: number of lines yielding unc-32::GFP insertions over total injected lines. (PDF) [file pgen.1009755.s002.pdf]

Strain	chrom	gene	PCR confirmation	cuts dpy-10	GFP inserts
**Strain 1**	**III**	***unc-119***	**+**	**+**	**1/2**
Strain 2	IV	*pes-1*	n.t.	+	3/7
Strain 3	V	Y69H2.3	+	+	0/6
**Strain 4**	**III**	**F53A2.9**	**+**	**+**	**5/5**
Strain 5	X	K05G3.1	n.t.	-	n.t.
Strain 6	II	*clec-119*	n.t.	n.t.	1/8
Strain 7	I	*eipr-1*	+	+	3/5
Strain 8	?	unmapped	-	+	**high**
Strain 9	V	F21D9.6	+	+	**low**
**Strain 10**	**I**	**W01A8.6**	**+**	**+**	**7/11**
Strain 11	?	unmapped	n.t.	n.t.	0/6
Strain 12	X	C23H4.6	n.t.	n.t.	n.t.
Strain 13	V	*srab-21*	n.t.	n.t.	0/1
**Strain 14**	**V**	**W03F9.11**	**+**	**n.t.**	**4/5**

S2 Table Cas9 insertions by miniMos. Summary of analyses of 14 Cas9 miniMos integration strains. ‘Strain’ is the isolation name. ‘chrom’ and ‘gene’ identify the location of the insert determined by inverse PCR, ‘unmapped’ means the inverse PCR failed or was ambiguous. PCR confirmation: ‘+’ indicates position was confirmed by locus-specific PCR, ‘-‘ indicates confirmatory PCR failed. ‘n.t.’, not tested. ‘cuts *dpy-10*’: P0s injected with a *dpy-10* sgRNA expressing plasmid, ‘+’ indicates the presence, and ‘-‘ indicates the absence of Dpy and Rol worms among the progeny of those P0s. ‘GFP inserts’: number of lines yielding *unc-32*::GFP insertions over total injected lines.
