# Supplementary material for: High-efficiency CRISPR gene editing in C. elegans using Cas9 integrated into the genome
Source: PLoS Genet. 2021 Nov 8;17(11):e1009755. doi: 10.1371/journal.pgen.1009755 (PMC8601624; doi:10.1371/journal.pgen.1009755)
Supplement: S3 Table — Bolded strains are the most useful for CRISPR modifications using unc-119 selections: EG9747 chromosome II, EG9881 chromosome III, EG9887 chromosome I, EG9891 chromosome V. (PDF) [file pgen.1009755.s003.pdf]

Strain	Genotype
N2	-
EG6249	*ttTi5605* II ; *unc-119*(ed3) III
none	*oxSi1196 [Pmex-5::Cas9(cDNA) :tbb-2 3'UTR Cbr-unc-119+; *ttTi5605] II ; unc-119(ed3)* III
EG9615	*oxSi1091[Pmex-5::cas9(+smu-2* introns*)::tbb-2 3'UTR unc-119+; *ttTi5605]* II; *unc-119(ed3)* III
**EG9747**	*oxSi1106[Pmex-5:Cas9:tbb-2 3'UTR +lox2272; *ttTi5605]* II ; *unc-119(ed3)* III
EG9814	*unc-119(ox819)* III
EG9809	*unc-119(ox819 oxTi1083[Pmex-5::cas9(+smu-2* introns*) + lox2272])* III
EG9873	*unc-119(ox819 oxTi1113[Pmex-5::cas9(+ smu-2* introns*), Phsp-16.41::Cre + lox2272 Cbr-unc-119 lox2272])* III
EG9874	*unc-119(ox819 oxTi1111[Pmex-5::cas9(+ smu-2* introns*), Phsp-16.41::Cre + lox2272])* III
EG9875	*unc-119(ox819 oxTi1122[Pmex-5::cas9(+ smu-2* introns*), Phsp-16.41::Cre, Pmyo-2::2xNLS-CyOFP + lox2272 Cbr-unc-119 lox2272])* III
EG9876	*unc-119(ox819 oxTi1126[Pmex-5::cas9(+ smu-2* introns*), Phsp-16.41::Cre, Pmyo-2::2xNLS-CyOFP + lox2272])* III
EG9877	*F53A2.9(oxTi1112[Pmex-5::cas9(+smu-2* introns*) + lox2272]), unc-119(ox819)* III
EG9878	*F53A2.9(oxTi1118[Pmex-5::cas9(+ smu-2* introns*), Phsp-16.41::Cre + lox2272 Cbr-unc-119 lox2272], unc-119(ox819)* III
EG9879	*F53A2.9(oxTi1118[Pmex-5::cas9(+ smu-2* introns*), Phsp-16.41::Cre + lox2272]), unc-119(ox819)* III
EG9880	*F53A2.9(oxTi1123[Pmex-5::cas9(+ smu-2* introns*), Phsp-16.41::Cre, Pmyo-2::2xNLS-CyOFP + lox2272 Cbr-unc-119 lox2272]), unc-119(ox819)* III
**EG9881**	*F53A2.9(oxTi1127[Pmex-5::cas9(+ smu-2* introns*), Phsp-16.41::Cre, Pmyo-2::2xNLS-CyOFP + lox2272]), unc-119(ox819)* III
EG9882	*F53A2.9(oxTi1127[Pmex-5::cas9(+ smu-2* introns*), Phsp-16.41::Cre, Pmyo-2::2xNLS-CyOFP + lox2272])* III
EG9883	*W01A8.6(oxTi1114[Pmex-5::cas9(+smu-2* introns*) + lox2272])* I ; *unc-119(ox819)* III
EG9884	*W01A8.6(oxTi1120[Pmex-5::cas9(+ smu-2* introns*), Phsp-16.41::Cre + lox2272 Cbr-unc-119 lox2272]* I ; *unc-119(ox819)* III
EG9885	*W01A8.6(oxTi1120[Pmex-5::cas9(+ smu-2* introns*), Phsp-16.41::Cre + lox2272])* I ; *unc-119(ox819)* III
EG9886	*W01A8.6(oxTi1124[Pmex-5::cas9(+ smu-2* introns*), Phsp-16.41::Cre, Pmyo-2::2xNLS-CyOFP + lox2272 Cbr-unc-119 lox2272])* I ; *unc-119(ox819)* III
**EG9887**	*W01A8.6(oxTi1128[Pmex-5::cas9(+ smu-2* introns*), Phsp-16.41::Cre, Pmyo-2::2xNLS-CyOFP + lox2272])* I ; *unc-119(ox819)* III
EG9888	*W01A8.6(oxTi1128[Pmex-5::cas9(+ smu-2* introns*), Phsp-16.41::Cre, Pmyo-2::2xNLS-CyOFP + lox2272])* I
EG9889	*unc-119(ox819)* III ; *W03F9.11(oxTi1116[Pmex-5::cas9(+smu-2* introns*) + lox2272])* V
EG9890	*unc-119(ox819)* III ; *W03F9.11(oxTi1121[Pmex-5::cas9(+ smu-2* introns*), Phsp-16.41::Cre + lox2272 Cbr-unc-119 lox2272]* V
**EG9891**	*unc-119(ox819)* III ; *W03F9.11(oxTi1121[Pmex-5::cas9(+ smu-2* introns*), Phsp-16.41::Cre + lox2272])* V
EG9892	*unc-119(ox819)* III ; *W03F9.11(oxTi1125[Pmex-5::cas9(+ smu-2* introns*), Phsp-16.41::Cre, Pmyo-2::2xNLS-CyOFP + lox2272 Cbr-unc-119 lox2272])* V
EG9893	*unc-119(ox819)* III ; *W03F9.11(oxTi1129[Pmex-5::cas9(+ smu-2* introns*), Phsp-16.41::Cre, Pmyo-2::2xNLS-CyOFP + lox2272])* V
EG9894	*W03F9.11(oxTi1129[Pmex-5::cas9(+ smu-2* introns*), Phsp-16.41::Cre, Pmyo-2::2xNLS-CyOFP + lox2272])* V

S3 Table Strains used in this study. Bolded strains are the most useful for CRISPR modifications using *unc-119* selections: EG9747 chromosome II, EG9881 chromosome III, EG9887 chromosome I, EG9891 chromosome V.
