# Supplementary material for: High-efficiency CRISPR gene editing in C. elegans using Cas9 integrated into the genome
Source: PLoS Genet. 2021 Nov 8;17(11):e1009755. doi: 10.1371/journal.pgen.1009755 (PMC8601624; doi:10.1371/journal.pgen.1009755)
Supplement: S4 Table — (PDF) [file pgen.1009755.s004.pdf]

Plasmid	Description	Availability	Reference
pMLS538	pDONR-221:2xNLS-Cas9(+*smu-2* introns)	Addgene	this study
pMLS539	pDONR-221:2xNLS-Cas9(cDNA + syntron)	-	this study
pMLS544	pEXPR(5605):P*mex-5::Cas9*(+*smu-2* introns)::*tbb-2* 3'UTR; *Cbr-unc-119*	Addgene	this study
pMLS546	pEXPR(5605):P*mex-5::Cas9*(cDNA + syntron)::*tbb-2* 3'UTR; *Cbr-unc-119*	Addgene	this study
pMLS389	pEXPR:P*U6::sgRNA(unc-119)U6*-terminator	-	this study
pMLS597	pEXPR:P*U6::sgRNA*(*dpy-10*)*U6*-terminator	Addgene	this study
pMLS640	pEXPR(5605):P*mex-5::Cas9*(+*smu-2* introns)::*tbb-2* 3'UTR; lox2272 *Cbr-unc-119* lox2272	Addgene	this study
pMLS621	pDEST(5605):lox2272 *Cbr-unc-119* lox2272	Addgene	this study
pMLS713	pDEST(miniMos):lox2272 *Cbr-unc-119* lox2272	Addgene	this study
pMLS714	pEXPR(5605):P*mex-5::Cas9*(+*smu-2* introns)::*tbb-2* 3'UTR ; lox2272 *Cbr-unc-119* lox2272	Addgene	this study
pMLS715	pDEST(miniMos-5'Arm):lox2272 *Cbr-unc-119* lox2272	Addgene	this study
pMLS716	pEXPR(miniMos-5'Arm):P*hsp16.41::Cre::tbb-2* 3'UTR ; lox2272 *Cbr-unc-119* lox2272	Addgene	this study
pMLS719	pEXPR:P*U6::sgRNA*(miniMos-5'Arm)*U6*-terminator	Addgene	this study
pMLS791	pEXPR:P*hsp16.41::Cre::tbb-2* 3'UTR ; P*myo-2::2xNLS-cyOFP::let-858* 3'UTR ; lox2272 *Cbr-unc-119* lox2272	Addgene	this study
pMLS338	pCRISPR:*unc-32::gfp + loxp Cbr-unc-119 loxp*	Addgene	Schwartz and Jorgensen, 2016
pMLS255	pCRISPR:*gfp::unc-17 + loxp Cbr-unc-119 loxp*	-	Schwartz and Jorgensen, 2016
pSYC33	pCRISPR:*snt-1::gfp + loxp Cbr-unc-119 loxp*	-	this study
pMLS357	pCRISPR:*sng-1::gfp + loxp Cbr-unc-119 loxp*	-	Schwartz and Jorgensen, 2016
pMLS276	pCRISPR:*gfp::snb-1 + loxp Cbr-unc-119 loxp*	-	Schwartz and Jorgensen, 2016
pMLS817	pCRISPR:*gfp::rab-3 + loxp Cbr-unc-119 loxp*	-	this study
pMLS485	pEXPR:P*eft-3::Cas9(+smu-2* introns)::*tbb-2* 3'UTR	Addgene	this study
pMLS1272	pEXPR:*ric-4 sgRNA*	Addgene	This study

Supplemental Table 4. Plasmids used in this study.
