# Supplementary material for: High-efficiency CRISPR gene editing in C. elegans using Cas9 integrated into the genome
Source: PLoS Genet. 2021 Nov 8;17(11):e1009755. doi: 10.1371/journal.pgen.1009755 (PMC8601624; doi:10.1371/journal.pgen.1009755)
Supplement: S1 Fig — The classic unc-119(ed3) allele was generated using EMS, and is likely to have other mutations in its background. Because CRISPR edits should be generated in a wild-type background that has not been exposed to a chemical mutagen, we generated a new unc-119 mutation in a wild-type background. N2 worms were injected with a plasmid encoding Cas9 and a guide RNA targeting the same region of unc-119 as ed3. Sequencing of ox819 revealed an 11 base-pair deletion starting at V110 (UNC-119a) straddling the targeted cut site which leads to a frameshift and stop after 27aa. (PDF) [file pgen.1009755.s005.pdf]

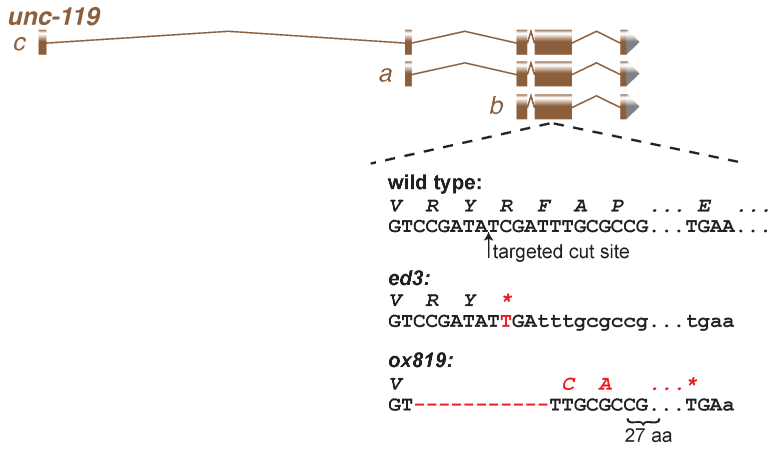

S1 Fig Generating *unc-119(ox819)*. The classic *unc-119(ed3)* allele was generated using EMS, and is likely to have other mutations in its background. Because CRISPR edits should be generated in a wild-type background that has not been exposed to a chemical mutagen, we generated a new *unc-119* mutation in a wild-type background. N2 worms were injected with a plasmid encoding Cas9 and a guide RNA targeting the same region of *unc-119* as *ed3.* Sequencing of *ox819* revealed an 11 base-pair deletion starting at V110 (UNC-119a) straddling the targeted cut site which leads to a frameshift and stop after 27aa.
