# Supplementary material for: High-efficiency CRISPR gene editing in C. elegans using Cas9 integrated into the genome
Source: PLoS Genet. 2021 Nov 8;17(11):e1009755. doi: 10.1371/journal.pgen.1009755 (PMC8601624; doi:10.1371/journal.pgen.1009755)
Supplement: S2 Fig — (Top) Fluorescence pattern of nuclear localized ‘cyan-excitable Orange Fluorescent Protein’ (cyOFP) in pharynx of an L4 larva from the Pmyo-2::2xNLS-cyOFP::let-858 3’UTR transgene (EG9882). Fluorescence is brightest in L3 and L4 larvae and is dim in adults. (Bottom) cyOFP has a broad excitation range (maximum from ~480–520) and orange fluorescence emission (590 nm peak) that can be observed using a mercury lamp and standard GFP longpass filter sets on a dissecting scope or using 488 nm or 514 nm lasers with an orange-red bandpass or long-pass emission filter (tagRFP, mScarlet, mRuby) on a confocal microscope. Emission and excitation spectra from fpbase.org. (PDF) [file pgen.1009755.s006.pdf]

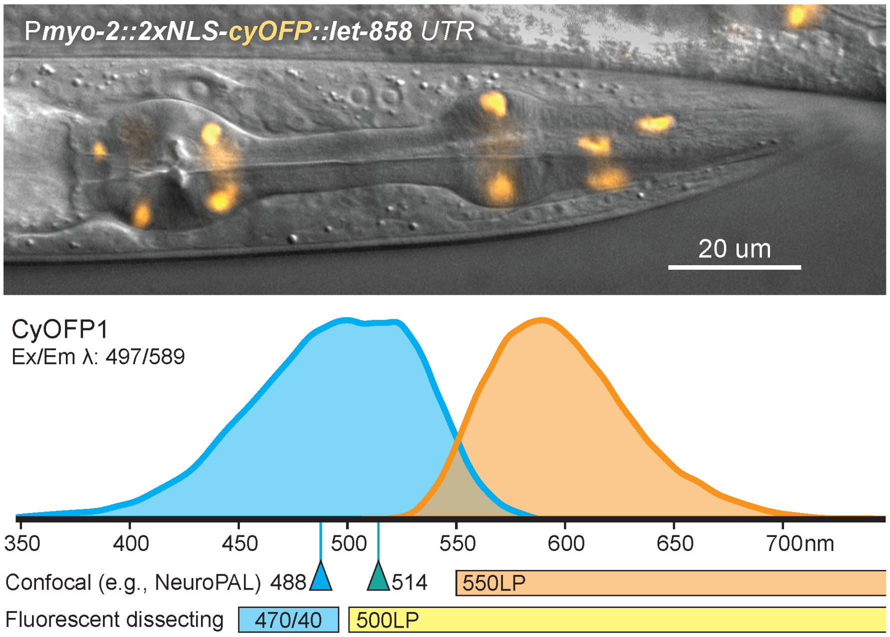


**S2 Fig Expression pattern of Cas9 locus fluorescent marker**. (Top) Fluorescence pattern of nuclear localized ‘cyan-excitable Orange Fluorescent Protein’ (cyOFP) in pharynx of an L4 larva from the P*myo-2::2xNLS-cyOFP::let-858 3’UTR* transgene (EG9882). Fluorescence is brightest in L3 and L4 larvae and is dim in adults. (Bottom) cyOFP has a broad excitation range (maximum from ~480-520) and orange fluorescence emission (590 nm peak) that can be observed using a mercury lamp and standard GFP longpass filter sets on a dissecting scope or using 488 nm or 514 nm lasers with an orange-red bandpass or long-pass emission filter (tagRFP, mScarlet, mRuby) on a confocal microscope. Emission and excitation spectra from fpbase.org.
