# Supplementary material for: High-efficiency CRISPR gene editing in C. elegans using Cas9 integrated into the genome
Source: PLoS Genet. 2021 Nov 8;17(11):e1009755. doi: 10.1371/journal.pgen.1009755 (PMC8601624; doi:10.1371/journal.pgen.1009755)
Supplement: S3 Fig — (A and B) Dead-end target sites: 20 EG9881 animals were injected with a guide RNA and repair template for tagging unc-32 with gfp. At starvation of the primary plates, array+, gfp- animals were singled to generate secondary plates, and the plates were incubated until starvation. Some lines failed to generate GFP+ animals (8/20 lines) by the time the secondary plate starved. These lines were analyzed by PCR amplification of the targeted insertion site (A) and then followed by sequencing (B). (A) PCR amplification of the unc-32 target locus. Expected amplicon sizes for the native locus and for a complete gfp + Cbr-unc-119 insert are indicated. (B) Sequencing results for the two unc-32 loci in each strain. (Top) the native unc-32 target site is shown with the guide RNA binding site and Cas9 cut site highlighted. For each strain, the sequences for both chromosomes are shown. Unique alleles are individually color coded to highlight sibling relationships. Strains 1, 2, 4, 6 and 7 likely originated from a single parent, and strains 3 and 8 likely originated from a single parent. These sequences document six different healing events: three chromosomes healed by NHEJ (red, blue, purple), one chromosome (green) incorporated a truncated GFP, one chromosome (brown) inserted 4 nucleotides at the cut site, and one chromosome (pink) inserted a novel segment with sequence variations (underlined). It is possible this sequence represents a de novo event segregating in this strain. (C) Location of two ‘dark inserts’. Two strains with off target insertions of unc-119(+) were located by whole genome sequencing (Illumina). After aligning all reads to the reference C. elegans genome, reads that failed to align to the genome were aligned to the sequences of all injected plasmid species. From this mapping, we determined which array sequences were present in the genome. To locate the junctions between the plasmid and the genome, we identified regions by inspection where the plasmid reads con [file pgen.1009755.s007.pdf]

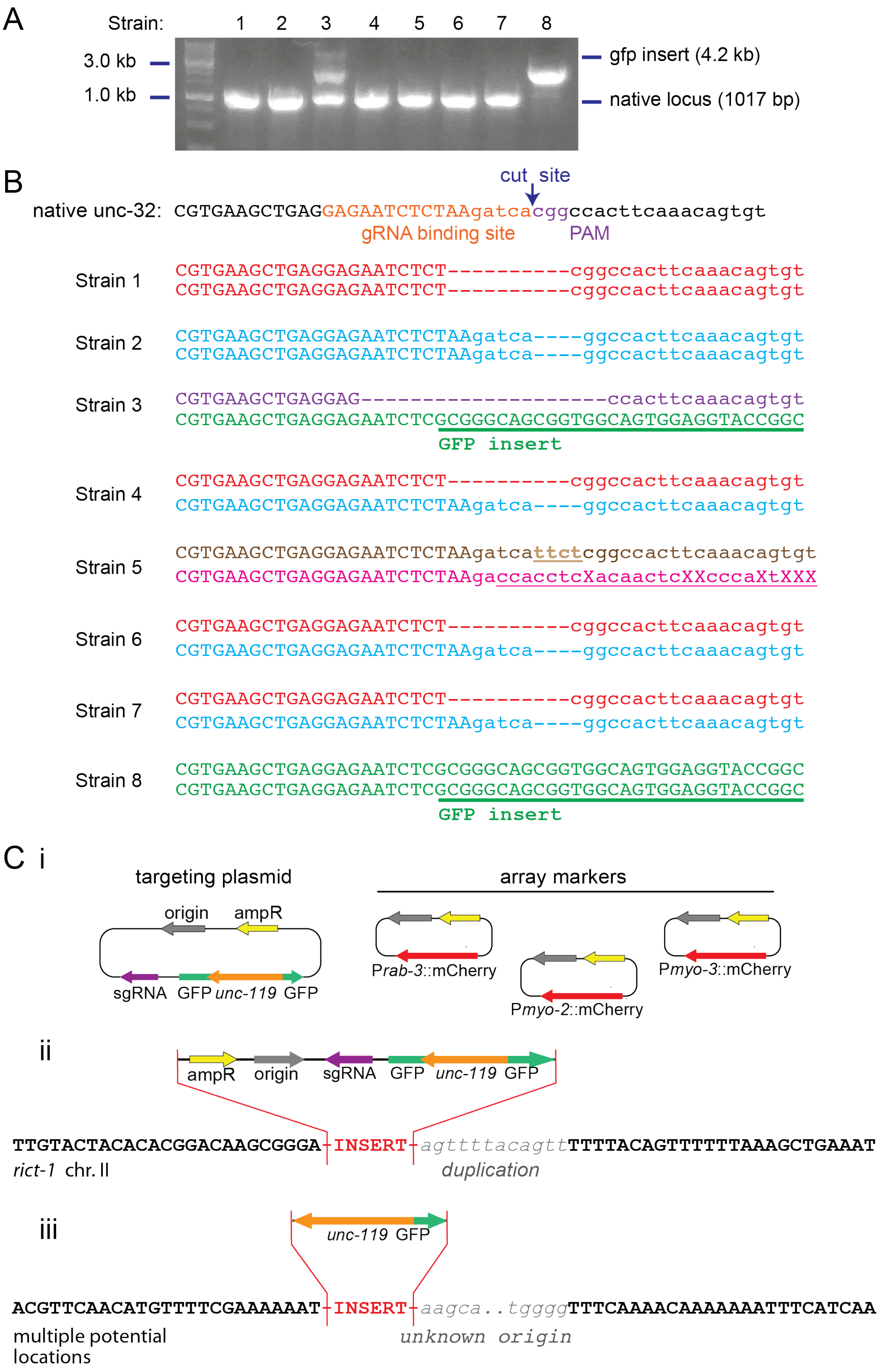
A
Strain:
1
2
3
4
5
6
7
8
3.0 kb
1.0 kb
gfp insert (4.2 kb)
native locus (1017 bp)
B
cut site
native unc-32: CGTGAAGCTGAGGAGAATCTCTAAgatcacggccacttcaaacagtgt
gRNA binding site
PAM
Strain 1
CGTGAAGCTGAGGAGAATCTCT-----------cggccacttcaaacagtgt
CGTGAAGCTGAGGAGAATCTCT-----------cggccacttcaaacagtgt
Strain 2
CGTGAAGCTGAGGAGAATCTCTAAgatca----ggccacttcaaacagtgt
CGTGAAGCTGAGGAGAATCTCTAAgatca----ggccacttcaaacagtgt
Strain 3
CGTGAAGCTGAGGAG--------------------ccacttcaaacagtgt
CGTGAAGCTGAGGAGAATCTCGCGGGCAGCGGTGGCAGTGGAGGTACCGGC
GFP insert
Strain 4
CGTGAAGCTGAGGAGAATCTCT-----------cggccacttcaaacagtgt
CGTGAAGCTGAGGAGAATCTCTAAgatca----ggccacttcaaacagtgt
Strain 5
CGTGAAGCTGAGGAGAATCTCTAAgatcattctcggccacttcaaacagtgt
CGTGAAGCTGAGGAGAATCTCTAAgaccacctcXacaactcXXcccaXtXXX
Strain 6
CGTGAAGCTGAGGAGAATCTCT-----------cggccacttcaaacagtgt
CGTGAAGCTGAGGAGAATCTCTAAgatca----ggccacttcaaacagtgt
Strain 7
CGTGAAGCTGAGGAGAATCTCT-----------cggccacttcaaacagtgt
CGTGAAGCTGAGGAGAATCTCTAAgatca----ggccacttcaaacagtgt
Strain 8
CGTGAAGCTGAGGAGAATCTCGCGGGCAGCGGTGGCAGTGGAGGTACCGGC
CGTGAAGCTGAGGAGAATCTCGCGGGCAGCGGTGGCAGTGGAGGTACCGGC
GFP insert
C i
targeting plasmid
origin
ampR
sgRNA
GFP
unc-119
GFP
array markers
Prab-3::mCherry
Pmyo-2::mCherry
Pmyo-3::mCherry
ii
ampR
origin
sgRNA
GFP
unc-119
GFP
TTGTACTACACACGGACAAGCGGGA
INSERT
agttttacagtt
TTTTACAGTTTTTTAAAGCTGAAAT
rict-1 chr. II
duplication
iii
unc-119
GFP
ACGTTCAACATGTTTTCGAAAAAAT
INSERT
aagca..tgggg
TTTCAAAACAAAAAAATTTCATCAA
multiple potential locations
unknown origin

S3 Fig Unintended CRISPR outcomes: dead-end targets and dark inserts.
(A and B) Dead-end target sites: 20 EG9881 animals were injected with a guide RNA and repair template for tagging *unc-32* with *gfp.* At starvation of the primary plates, array+, *gfp-* animals were singled to generate secondary plates, and the plates were incubated until starvation. Some lines failed to generate GFP+ animals (8/20 lines) by the time the secondary plate starved. These lines were analyzed by PCR amplification of the targeted insertion site (A) and then followed by sequencing (B). **(A)** PCR amplification of the *unc-32* target locus. Expected amplicon sizes for the native locus and for a complete *gfp+ Cbr-unc-119* insert are indicated. **(B)** Sequencing results for the two *unc-32* loci in each strain. The native *unc-32* target site is shown above with the guide-RNA binding site and Cas9 cut site highlighted. For each strain, the sequences for both chromosomes are shown. Unique alleles are individually color coded to highlight sibling relationships. Strains 1, 2, 4, 6 and 7 likely originated from a single parental, and strains 3 and 8 likely originated from a single parent. These sequences document six different healing events: three chromosomes healed by NHEJ (red, blue, purple), one chromosome (green) incorporated a truncated GFP, one chromosome (brown) inserted 4 nucleotides at the cut site, and one chromosome (pink) inserted a novel segment with sequence variations (underlined). 'X' indicates that two nucleotides were present at this position. It is possible this sequence represents a *de novo* event segregating in this strain.
(C) Location of two 'dark inserts'. Two strains with off target insertions of *unc-119(+)* were located by whole genome sequencing (Illumina). After aligning all reads to the reference *C. elegans* genome, reads that failed to align to the genome were aligned to the sequences of all injected plasmid species. From this mapping, we determined which array sequences were present in the genome. To locate the junctions between the plasmid and the genome, we identified regions by inspection where the plasmid reads contained unmapped tails. BLAST analysis of the unmapped tails was used to determine the insertion site. The insertion site of one of the off-target events is in a central intron of the *rict-1* gene. The sequences flanking the second off-target insertion are present at multiple loci in the genome, preventing exact mapping. **(i)** Schematic representation of the CRISPR targeting injection mix containing a targeting plasmid and multiple array marker plasmids. **(ii)** Schematic representation of *rict-1(ox773),* an off-target insertion of the entire *gfp::snb-1* targeting plasmid into intron #9 of *rict-1a* on chromosome II. **(iii)** Schematic representation of the off-target insertion of a portion of the *unc-32::gfp* targeting plasmid into a non-unique genomic location. The insertions were not at sites obviously similar to the predicted guide RNA binding sites.
