# Supplementary material for: High-efficiency CRISPR gene editing in C. elegans using Cas9 integrated into the genome
Source: PLoS Genet. 2021 Nov 8;17(11):e1009755. doi: 10.1371/journal.pgen.1009755 (PMC8601624; doi:10.1371/journal.pgen.1009755)
Supplement: S4 Fig — Insertion sites of the integrated Cas9 alleles (red) in the genome. The locus into which the miniMos-Cas9 transposon inserted is written above (for example ‘W01A8.6’), and the original, unmodified, transposon insertions are named (‘oxTi’). oxSi1106 is a single-copy insertion at the Mos1 insertion site ttTi5605. Also depicted are prominent MosSCI sites (light blue) and ‘universal MosSCI ‘sites (dark blue) [26,27]. ‘Universal MosSCI’ sites use the ttTi5605 arms for homologous recombination insertion and are usually flanked by NeoR and unc-18(+) transgenes (see www.wormbuilder.org website). (PDF) [file pgen.1009755.s008.pdf]

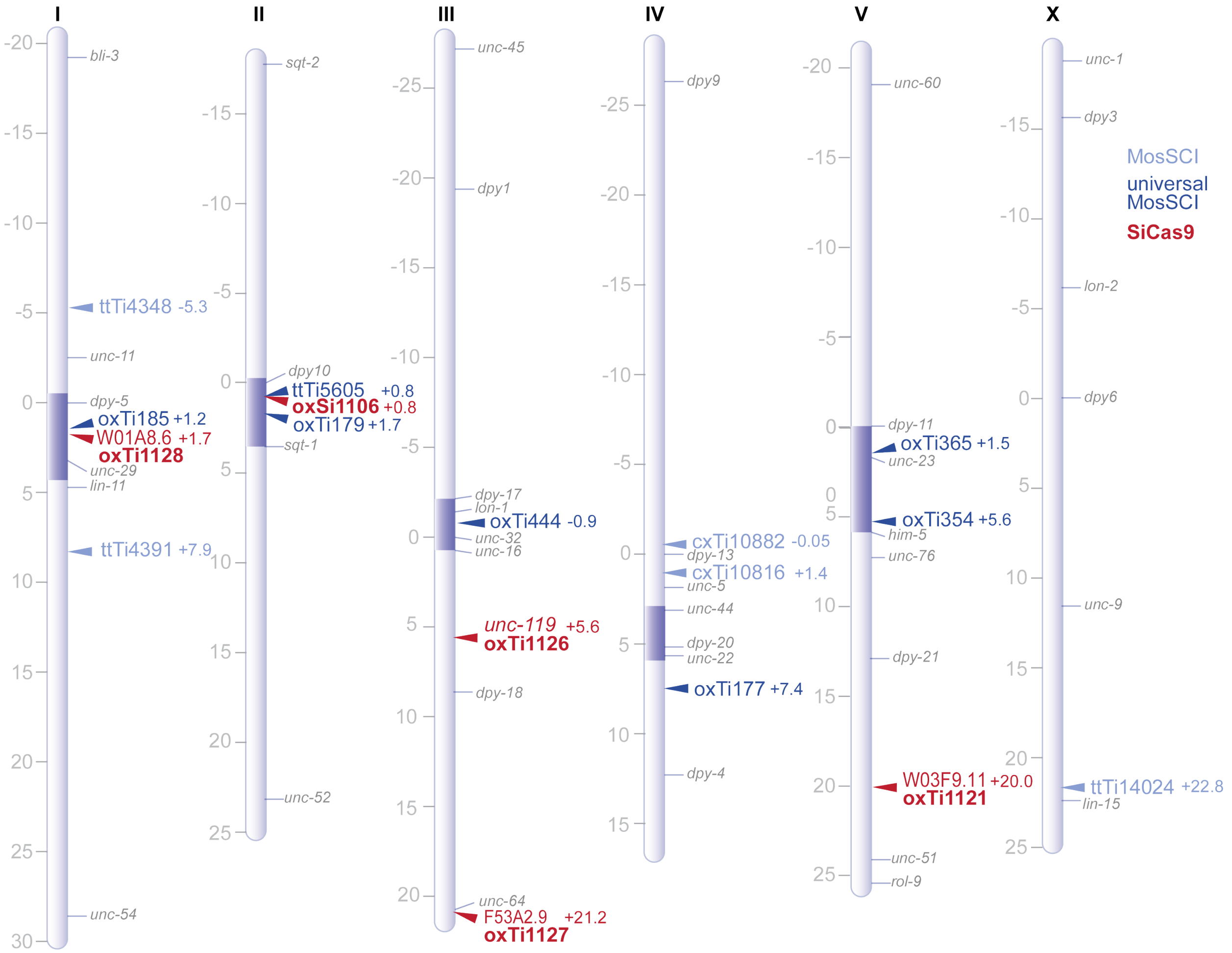

S4 Fig Genetic map of Cas9 and MosSCI target sites. Insertion sites of the integrated Cas9 alleles (red) in the genome. The locus into which the miniMos-Cas9 transposon inserted is written above (for example 'W01A8.6'), and the original, unmodified, transposon insertions are named ('*oxTi*'). *oxSi1106* is a single-copy insertion at the Mos1 insertion site *ttTi5605*. Also depicted are prominent MosSCI sites (light blue) and 'universal MosSCI 'sites (dark blue) (Frøkjær-Jensen et al., 2014, 2012). 'Universal MosSCI' sites use the *ttTi5605* arms for homologous recombination insertion and are usually flanked by NeoR and *unc-18(+)* transgenes (see www.wormbuilder.org website).
