# Supplementary material for: High-efficiency CRISPR gene editing in C. elegans using Cas9 integrated into the genome
Source: PLoS Genet. 2021 Nov 8;17(11):e1009755. doi: 10.1371/journal.pgen.1009755 (PMC8601624; doi:10.1371/journal.pgen.1009755)
Supplement: S5 Fig — Insertion sites are ordered by chromosome number (‘Chr’) and site into which the miniMos inserted (red). Strain and name of modified miniMos element are provided below (black). The chromosome II site is a MosSci insertion into the ttTi5605 site and does not have a Cre or orange fluorescent protein (‘cyOFP’) transgene. (PDF) [file pgen.1009755.s009.pdf]

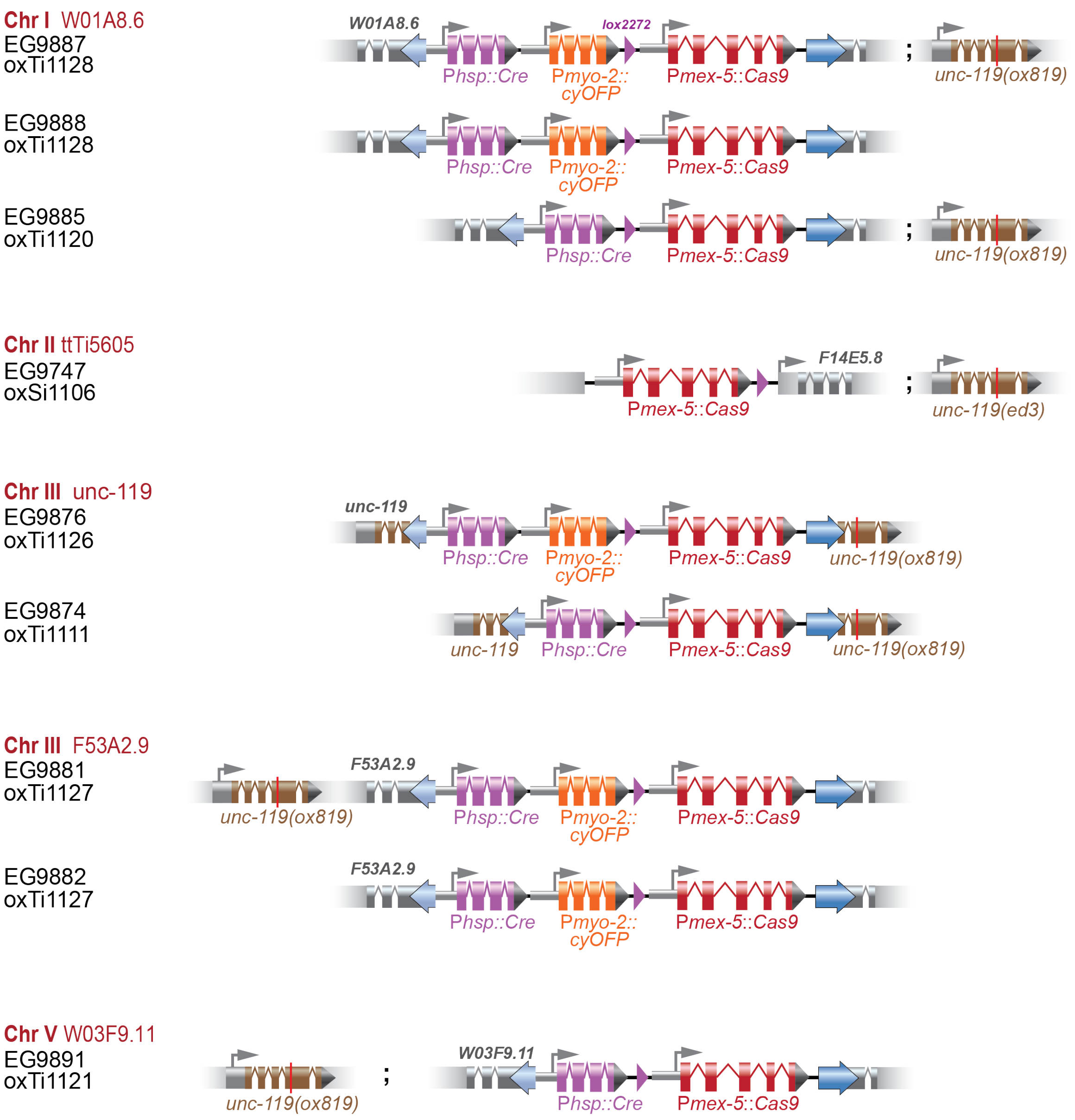

S5 Fig Gene models for Cas9 loci. Insertion sites are ordered by chromosome number (‘Chr’) and site into which the miniMos inserted (red). Strain and name of modified miniMos element are provided below (black). The chromosome II site is a MosSci insertion into the ttTi5605 site and does not have a Cre or orange fluorescent protein (‘cyOFP’) transgene.
