# Supplementary material for: High-efficiency CRISPR gene editing in C. elegans using Cas9 integrated into the genome
Source: PLoS Genet. 2021 Nov 8;17(11):e1009755. doi: 10.1371/journal.pgen.1009755 (PMC8601624; doi:10.1371/journal.pgen.1009755)

High-efficiency CRISPR gene editing in *C. elegans* using Cas9 integrated into the genome

Matthew L. Schwartz, M. Wayne Davis, Matthew S. Rich, and Erik M. Jorgensen*

Supplemental File 1

Annotated protocol for CRISPR gene editing in *C. elegans*

CRISPR protocol [1]

Injection:

Day 0 Grow appropriate Cas9-expressing strain at 15°C [2].

Day 1 Make an injection mix containing 100 ng/ul total DNA [3]:

Component	Amount (ng/ul)
Guide RNA expression plasmid [4,5]	30-90
Repair template [6]	10-30
Fluorescent markers [7]	10-20
Negative selection markers [8]	10
Water	To 100 ng/ul

Inject 10-20 animals [9].

Place 1-3 injected animals on individual HB101 plates.

Day 1-7 Incubate at 20°C until starvation [10].

Screening for potential inserts:

Day 8 Screen visually for phenotypically wild-type animals (*unc-119+)* [11] that have no fluorescent array markers. Alternatively, activate an inducible negative selection marker on the array and screen for resistant animals. Clone candidates (1-3 from each P0 plate with candidates) to new plates.

Isolation of *bona fide* inserts:

Day 8-10 Culture candidates at 20°C for one generation and inspect progeny for the *unc-119(+)* phenotype. Discard all plates that do not exhibit Mendelian ratios (*i.e.,* offspring are not ~75% or 100% wild type) [12].

Clone 12 rescued animals to new plates and incubate at 15°C to isolate homozygous lines [13]. Keep plates that throw 100% or near 100% *unc-119(+)* progeny as potential edits.

Day 10 Genotype lines for correct repair by PCR and sequencing [14].

Marker excision:

Day 11 Pick 5-10 larvae from a homozygous, PCR-verified line to a new plate and heat-shock at 32°C for 2 hours to express Cre recombinase.

Day 11-14 Remove animals from heat shock and culture at 25°C. Isolate *unc-119(-)* mutants from the progeny of the heat-shocked animals [15].

Outcross:

Day 21 Cross N2 males to the *unc-119(-)* insert(+) strain.

Day 25 Select F2 animals that have lost the cyOFP-marked Cas9 locus and are homozygous for the genome modification. Homozygose the *unc-119(+)* locus [16].

CRISPR protocol notes:

1 See CRISPR protocol illustrated steps (Protocol Fig.1)

2 Choose a strain in which the Cas9 insertion is not linked to your editing target. We isolated four high-efficiency Cas9 insertions on three chromosomes (Protocol Fig.2). These are available with or without *unc-119(-)* in the background (Protocol Fig.3). If your edit is not on chromosome III, we recommend using EG9881, which is the most efficient editing strain. Otherwise, we use EG9887, which in our hands is nearly as efficient as EG9881. Note that EG9893 does not have P*myo-2*::CyOFP marking the Cas9 locus.

**Strain**	**Cas9 locus**
EG9887	LGI +1.7
EG9876	LGIII +5.6
EG9881	LGIII +21.2
EG9893	LGV +20.0

3 Make high quality plasmid DNA preps using the Invitrogen Purelink kit or by ethanol precipitating plasmids from other kits.

4 We prefer cloning guide RNA expression cassettes and repair templates in separate plasmids since cloning them separately is more efficient and modular (e.g., the sample guide RNA plasmid can be used for multiple tags). We use SapTrap assembly (Schwartz and Jorgensen, 2016), and generally insert guide RNAs into pMLS134 (Addgene #73714) and repair templates into pMLS257 (Addgene # 73716).

5 In our hands, guide RNA expression is the limiting factor for editing efficiency. We therefore maximize the amount of guide RNA expression plasmid in the injection mix, typically using 60 ng / uL guide RNA expression plasmid.

6 Use 30 ng / uL repair template plasmid if using separate plasmids for guide RNA and template (e.g., pMLS134 and pMLS257). Use 90 ng / uL of a combined repair template and guide RNA expression plasmid (e.g., pMLS256). We have successfully edited with as low as 2 ng / uL repair template.

7 Fluorescent markers are required to identify successful injections and to track array positive animals in the population. We usually use a combination of pGH8 (Prab-3::mCherry, at 4 ng/ul), pCFJ90 (Pmyo-2::mCherry, at 2 ng/ul), and pCFJ104 (Pmyo-3::mCherry, at 4 ng/ul)

8 Negative selection markers are optional and assist in screening for loss of the array in successfully-injected animals. We often use pMA122 (*Phsp::peel-1)* or pNP403 (*Primb-1::histamine-gated chloride channel)* for negative selection.

9 Another limiting factor in recovering edits is injection quality. While injections do not need to be perfect, good injections that yield 20+ array(+) F1s are usually sufficient to yield edited lines. But adjust the number of injected animals based on your injection skills.

10 CRISPR modified worms appear as early as the F_2 generation. If using a negative selection marker on the array, wait until starvation or near starvation to maximize the worm population before activating the selectable marker by heat-shock for pMA122 or histamine application for pNP403.

11 This strategy uses *unc-119* rescue to select for edited animals, making screening before the F_2 generation unnecessary. Because *unc-119* mutants grow slowly and cannot enter dauer, edited animals will be selected over the course of many generations. Although we usually screen for inserts after 2-3 generations, propagating rescued animals will increase the proportion of animals with an integrated *unc-119(+)* cassette.

12 Occasionally, we isolate “dark arrays” in which the animal is rescued by an array that has not incorporated the fluorescent markers. These generally produce far less than 75% rescued progeny, and should be discarded. Stable dark arrays that mimic Mendelian ratios can be identified by genotyping PCR.

13 The heat-shock Cre construct exhibits a low level of activity independent of heat-shock. Such events may lead to the mischaracterization of homozygous lines as heterozygous. Culturing strains at 15°C minimizes inappropriate excision of the selectable marker.

14 PCR will expose ‘dark inserts’, which lack a correctly inserted GFP. We usually use a three-primer PCR to genotype inserts, consisting of either oMLS1275 (5’- cgtcagagaggagaggaacg, binds in the *unc-119* promoter) or oMLS1276 (5’- caataattcctgtctagtttgttccttc, binds in the *unc-119* 3’UTR) and two primers that flank the insert site. Take care to design primers that will yield two differently-sized amplicons: one for the unedited genome and one for the genome + insertion. We usually have good success designing genomic primers with software like Primer3.

15 Generally, ~50% of the brood will be *unc-119* mutants resulting from marker excision. *unc-119(-)* animals grow more slowly than the wild type, so it may be necessary to perform egg lays to limit the total number of F1 progeny on plate.

16 The genome modification can be tracked during the outcross in a number of ways. Optimally, you can see it by fluorescence microscopy on a dissecting or compound fluorescence microscope. Alternatively, instead of crossing to N2 males, you can cross to males of a fluorescent balancer and screen for balancer(-) F2s. In the case that the edit requires detection by PCR, design the assay such that heterozygous and homozygous animals can be readily distinguished.

Protocol Figure 1. CRISPR protocol for *C. elegans* illustrated steps

1. starting strain

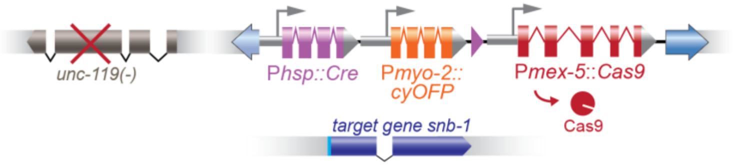

Day 0: grow at 15°C on HB101 for optimal injection quality.

2. inject DNA

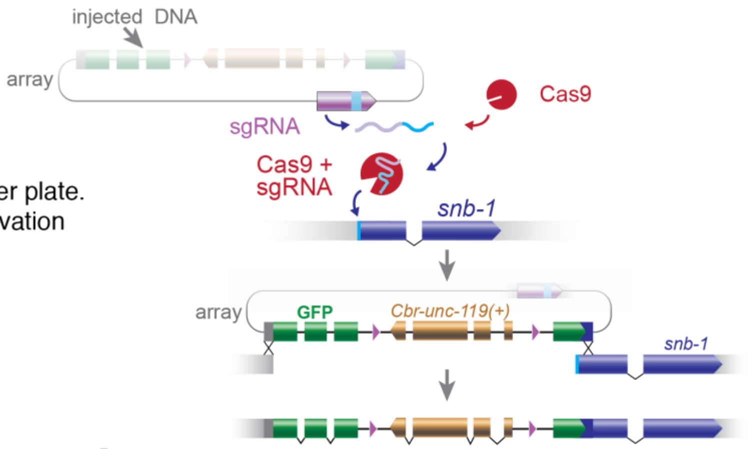

Day 1: Inject 10-20 P0s. Pool 2-3 injected animals per plate.
Day 2: Incubate at 18-20°C until plates are near starvation (~2-3 generations).

3. select non-Unc, array(-) progeny

Day 8: Screen plates for *unc-119*(+) and array(-) animals. Clone up to 2 animals from each plate to new HB101 plates.
Day 8-10: Incubate at 15°C to homozygose *unc-119*(+) tag.
Day 10: Genotype homozygous strains by GFP PCR and sequencing to confirm correct repair.

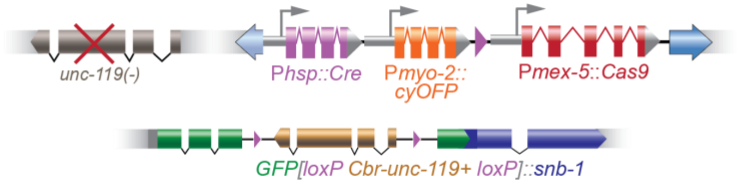

4. heat shock to excise selection

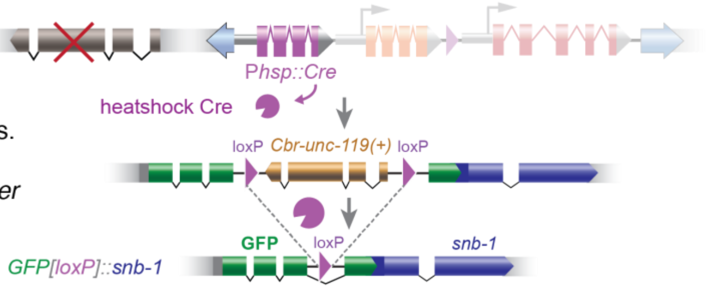

Day 11: Pool 20 L1-L2 animals and heatshock at 32°C for 2 hours.
Day 11-14: Incubate heatshocked plates at 15°C
Day 14: Isolate *unc-119*(-) animals. *NB: these animals grow slower than wildtype.*

5. outcross unc-119(-) and Cas9

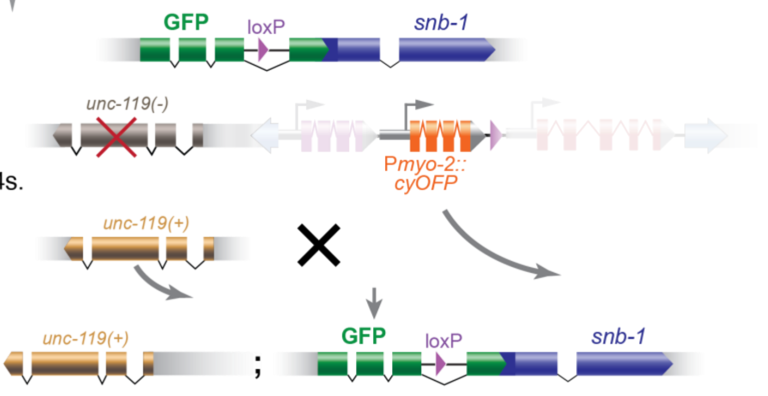

Day 21: Cross N2 or balancer males to GFP(+) *unc-119*(-) L4s.
Day 25: Clone GFP(+) unc-119(+) CyOFP(-) animals. *Enjoy!*

Protocol Figure 2. Genetic map of integrated Cas9 sites and MosSCI sites in *C. elegans*

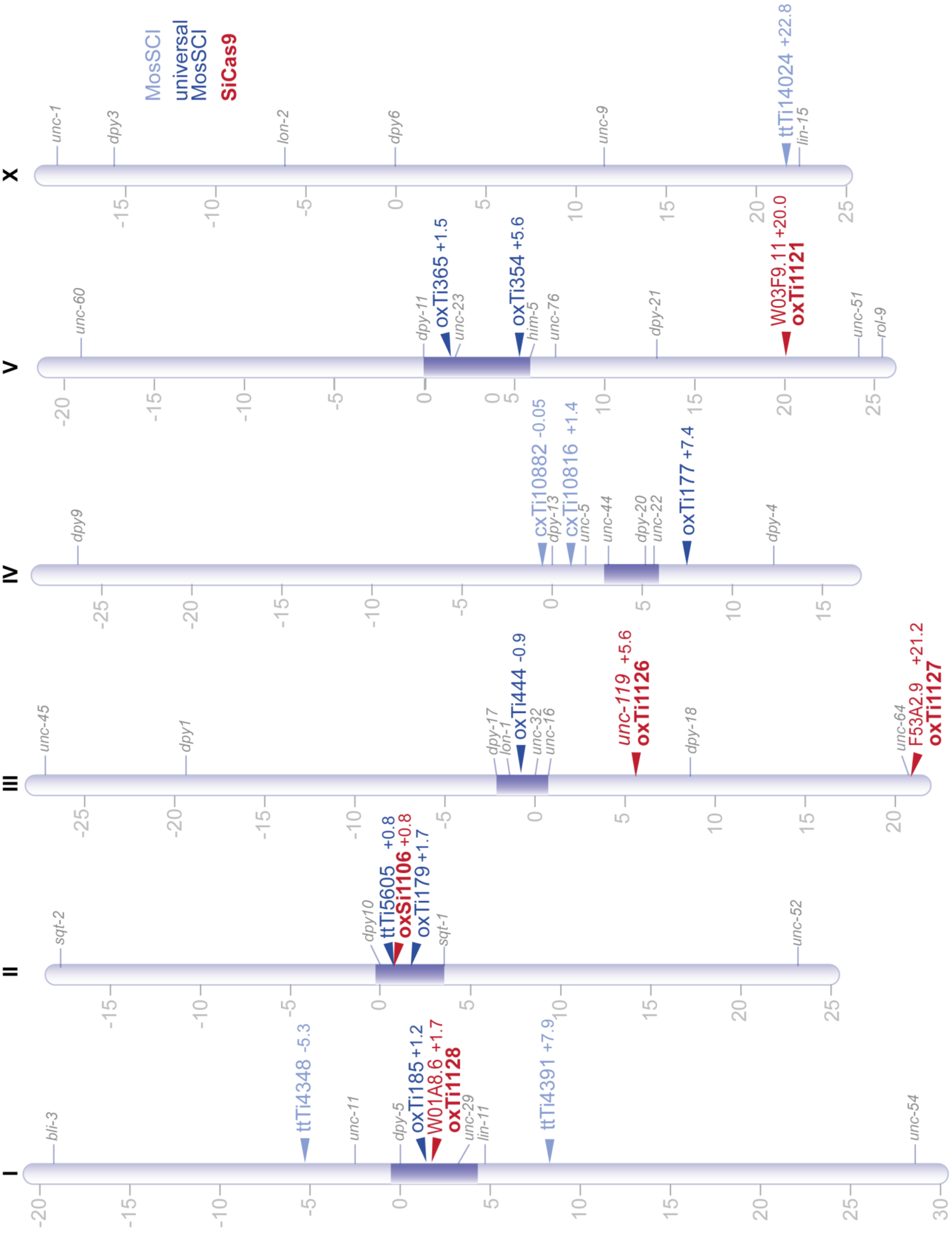

Protocol Figure 3. Integrated Cas9 loci details

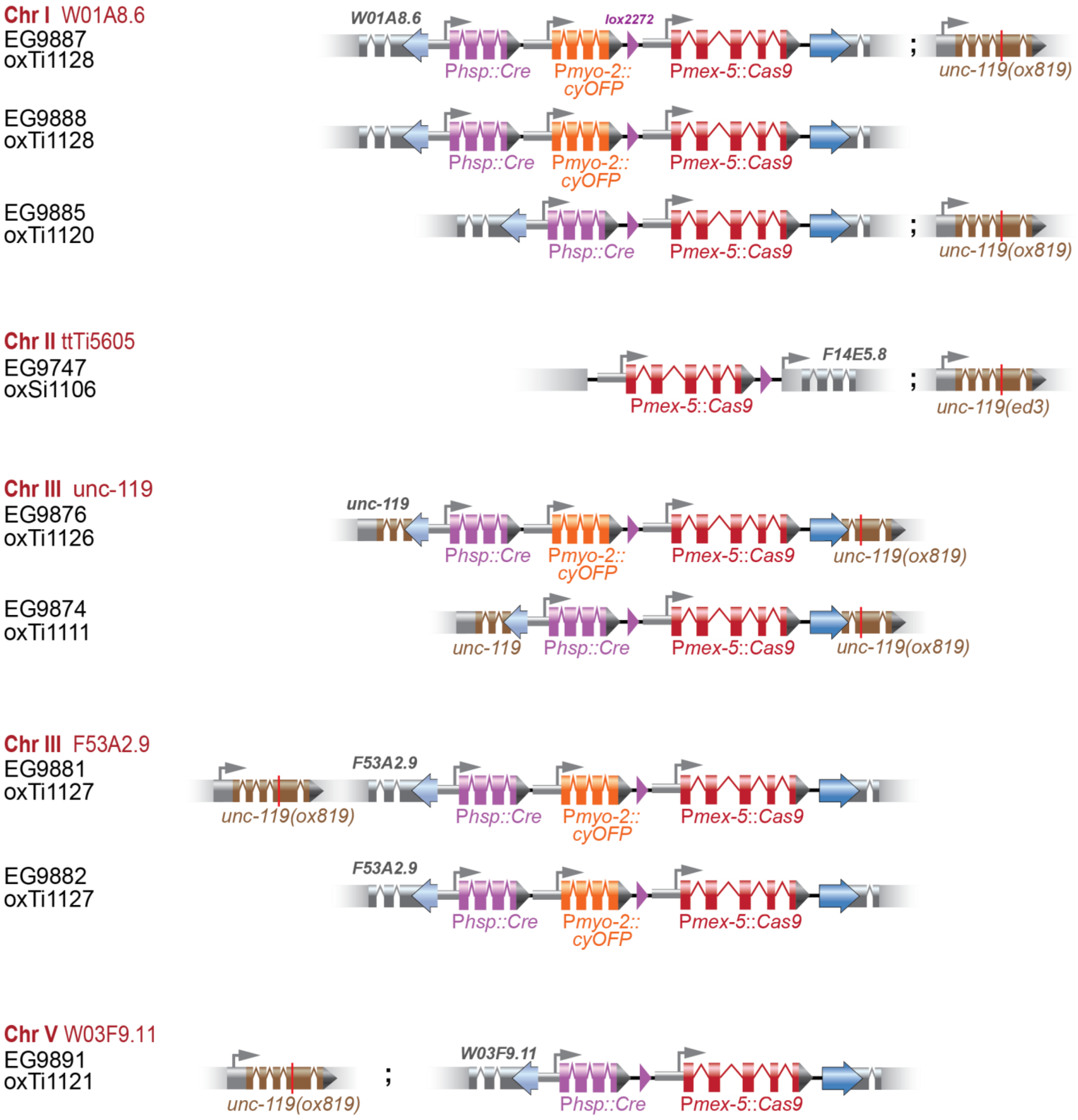

Supplement: S1 File — (PDF) [file pgen.1009755.s010.pdf]
